# Digital Twins of the Water Cooling System in a Power Plant Based on Fuzzy Logic

**DOI:** 10.3390/s21206737

**Published:** 2021-10-11

**Authors:** Carlos Antonio Alves de Araujo Junior, Juan Moises Mauricio Villanueva, Rodrigo José Silva de Almeida, Isaac Emmanuel Azevedo de Medeiros

**Affiliations:** 1 Centrais Elétricas da Paraíba, EPASA, João Pessoa 5045, PB, Brazil; carlosaaajr@gmail.com (C.A.A.d.A.J.); rodrigojsa@gmail.com (R.J.S.d.A.); isaac.medeiros@cear.ufpb.br (I.E.A.d.M.); 2 Renewable and Alternatives Energies Center (CEAR), Electrical Engineering Department (DEE), Campus I, Federal University of Paraiba (UFPB), Joao Pessoa 5045, PB, Brazil

**Keywords:** digital twins, power plants, cooling system, fuzzy logic

## Abstract

In the search for increased productivity and efficiency in the industrial sector, a new industrial revolution, called Industry 4.0, was promoted. In the electric sector, power plants seek to adapt these new concepts to optimize electric power generation processes, as well as to reduce operating costs and unscheduled downtime intervals. In these plants, PID control strategies are commonly used in water cooling systems, which use fans to perform the thermal exchange between water and the ambient air. However, as the nonlinearities of these systems affect the performance of the drivers, sometimes a greater number of fans than necessary are activated to ensure water temperature control which, consequently, increases energy expenditure. In this work, our objective is to develop digital twins for a water cooling system with auxiliary equipment, in terms of the decision making of the operator, to determine the correct number of fans. This model was developed based on the algorithm of automatic extraction of fuzzy rules, derived from the SCADA of a power plant located in the capital of Paraíba, Brazil. The digital twins can update the fuzzy rules in the case of new events, such as steady-state operation, starting and stopping ramps, and instability. The results from experimental tests using data from 11 h of plant operations demonstrate the robustness of the proposed digital twin model. Furthermore, in all scenarios, the average percentage error was less than 5% and the average absolute temperature error was below 3 °C.

## 1. Introduction

Brazil has diversified energy generation, mainly organized into hydroelectric, solar, wind, biomass, and thermoelectric plants. Specifically, this last source was used to increase energy security in the national interconnected system, and is widely used to supply energy to isolated systems [1]. In the last five years, power plants were responsible for an average of 19% of the energy generated in Brazil. This is due to the insertion of intermittent renewable sources, such as solar and wind, acting to stabilize the variability in generation in the short-term. In addition, thermoelectric plants operate significantly during dry periods, as the Brazilian energy organization is mostly hydroelectric.

Power plants need to maintain high operating performance, generation, availability, and reliability, in addition to reducing fuel costs and, at the same time, maintain low carbon emissions into the atmosphere. The specific consumption measure—the ratio between power and kilograms of fuel—is an indicator of thermal efficiency, but there is also another important measure that will be addressed in this work: the energy consumption of auxiliary equipment [2]. This equipment plays the function of ensuring that the parameters of the main devices (e.g., boilers, engines, generators, and turbines) responsible for the production of energy are controlled and, therefore, consume electricity from the production itself to maintain its operation [3].

Auxiliary equipment are those that have an auxiliary function to the operations of diesel machines, such as water pumps and lubricating oil in general, electric actuators, radiator fans, and air compressors. The activation of such equipment occurs through directly starting electric motors, which, without smooth current and voltage control, increase energy consumption. The electrical configuration of the power plant is made to operate at its highest generating capacity; however, at times, the generation is reduced, causing the auxiliary equipment to work in inefficient areas, as informed by the manufacturers [3].

A water cooling system is auxiliary equipment that, generally, comprises the system with the highest consumption in a plant. For example, in a large plant with a generation of 340 MW, the consumption of a refrigeration system reaches 5% of the total energy produced, which is a significant value. The cooling system consists of a closed water circuit, with several fans that are responsible for removing the heat from the water in the diesel generator’s cooling circuit. In some plants, the cooling system is shared between several generating units and the direct starting fans, and it is not possible to control the temperature based on variation of the fan speed. Likewise, the sharing of refrigeration makes the system nonlinear and dependent on several parameters associated with different generating units.

To reduce the inefficient operation of these systems, classic controller designs, such as proportional-integral-derivative (PID), are generally implemented in power plants; however, due to the nonlinear and time-invariant characteristics of the process, this can lead to a loss of performance of the controller and limits the efficiency of the process, thus losing tune during the operation (i.e., the loss of optimization of the dynamic characteristics of the desired response, such as damping, over-signal, accommodation time, and steady-state error). Therefore, in many industrial installations, PID controllers are not fine-tuned and, in some cases, are found operating in manual mode, due to the need for constant adjustments and readjustments.

The digital transformation of power plants allowed for the collection and storage of big data from the installation of multiple sensors and data acquisition systems, making the monitoring and diagnosis of machines a challenging task [4,5]. The convergence of the physical and virtual worlds of a thermal power plant is one of the main challenges of Industry 4.0, directing research to build more efficient and intelligent models and, thus, envisioning opportunities for the development of research on digital twins [6,7]. In the context of Industry 4.0, this work has the goal of enabling autonomous decision-making processes to increase the operational efficiency of the power plant. Furthermore, Industry 4.0 is based on integration of the information technology (IT) environment with industrial automation technology (AT) to make the environment more interactive between people, machines, and products in real-time, from which a cyber-physical system (in this work, the Digital Twin model) can be developed.

Digital Twin is a concept that creates models of physical systems with the ability to continuously adapt to changes in the environment or operation, using data from the sensors in real-time. Among the advantages of developing a digital twin for a machine is the ability to identify possible problems in the associated real, physical system. Therefore, the digital transformation in a power plant became a key point for the operation of an intelligent plant, providing decisive information and connections to support decision making in an industrial environment.

In this work, the objective is to develop digital twins of a water cooling system in a thermoelectric plant, based on fuzzy inference systems, to incorporate improvements in the energy efficiency of the cooling system. This computational model can process a large amount of data, relating internal and external parameters of the system for the automatic generation of rules from a set of numerical data. To validate this methodology, data from a thermoelectric plant located in the city of João Pessoa, Brazil, are used. The data correspond to a day of operation of the plant, where measurements of the power of the fans, controlled temperature, room temperature, and number of fans were recorded.

The main contributions of this work are: (a) the development of digital twins for the shared hot and cold water cooling system of five internal combustion engines; (b) methodology for the reduction of dimensionality using principal component analysis, making the computational system simple and flexible; (c) methodology that enables digital twins to have the ability to automate learning from new measurements, incorporating knowledge into the system through the automatic generation of new rules; and (d) providing a decision support tool for operators, allowing for improvements in the energy efficiency of the refrigeration system through the approximate analysis of systems which are very complex and nonlinear, making the application of conventional mathematical modeling techniques difficult.

## 2. Related Works

In the search for operational optimization, the industrial sector and companies were looking for new modern tools that are capable of contributing to increasing the efficiency of the plants, either assisting in operational decision making to reduce fuel consumption or assisting in the maintenance of equipment, to minimize equipment downtime. For this, the energy industry relies on techniques and concepts developed in the so-called fourth industrial revolution—Industry 4.0—incorporating the development of Digital Twins into its structure, as well as taking advantage of big-data analytics and the use of intelligent sensor, internet of things, and artificial intelligence technologies.

The creation of plant models usually involves interdisciplinary knowledge, involving specific areas of the problem in question, related to process control, signal processing, adaptive systems, and machine learning, among others. In this type of application, knowledge about the problem can be extracted through measurements from sensors and/or from the experience of human specialists, thus building a knowledge base for the construction of intelligent models that follow changes in the environmental behavior of physical systems.

The development of a digital twin involves standardization-based modeling, data fusion, and big data analytics; however, one form of implementation may differ from one application to another. In the literature, several solutions can be found for the design and implementation of digital twins, which can be developed for simple and complex systems. The methods used can be based on Artificial Neural Networks (ANNs), deep transfer learning, and rules-based expert systems [8,9,10,11]. Other practices and mathematical models to build digital twins of real systems for the development of system lifetime prediction systems were described in [12].

Many real-world applications require the construction of models through the use of input-output samples. In this context, systems based on fuzzy logic were used due to their ability to be used as universal approximators, in addition to being able to explain how their outputs are generated from the input values [13].

In [14,15], a general method was developed for the automatic extraction of fuzzy rules from the combination of information from numerical data and linguistic information, in a unified platform. This procedure was able to approximate continuous functions, being applied to time-series forecasting problems. To improve this procedure, in [16], optimization methodologies of the rule extraction method based on genetic algorithms (GAs) were used, being able to optimize the number of fuzzy sets and the position of the pertinence functions of the triangular type.

Genetic Algorithms (GAs) are global optimization techniques based on the principles of genetics and natural selection [17]. This new algorithm was tested in the optimization of electrical distribution networks in Spain, and its procedures were improved through the use of variable selection algorithms.

ANNs can be applied for the intelligent detection of failures in complex processes and systems, such as power plants, propulsion systems, rotating machinery, and gas turbine engines, among others. In this context, the application of an ANN aims to provide computational diagnostic tools to identify and classify between normal or fault operating conditions [18]. However, many of these diagnostic systems are black boxes, making it difficult to explain the results and extract knowledge, as this information is encoded in the weights of the ANN during its construction. To resolve these limitations, algorithms for the construction of knowledge and extraction of rules from an ANN, such as the KT rule extraction algorithm [19,20], subset algorithm [21], validity internal analysis (VIA) [22], rule-extraction-as-learning technique based on ANN [23,24], and so on, were developed. Although complementary algorithms can be applied to explain the results, large volumes of data are still needed to build valid examples to train this type of model.

In [25,26], methods for extracting fuzzy rules from training based on SVMs (Support Vector Machines) for multiclass classification problems were proposed. SVMs have several applications in classification and regression, and showed excellent performance in conceptual generalization. This method was used to address the problem of limitation in the interpretation of data input-output mapping, in which the model is usually treated as a black box.

In [27], an approach was introduced to implement a digital twin for industrial processes using fuzzy logic for the extraction of rules and artificial neural networks to learn knowledge from obtained data. This architecture was used in an industrial process, showing that digital twins can explore new possibilities in process automation and condition monitoring, real-time simulations, and maintenance forecasting, among others.

## 3. Cooling System Description

In this section, considerations related to the thermoelectric plant under study are presented, such as the characteristics of the water cooling system, auxiliary equipment, the variables involved in the process, and how they influence the nonlinear properties and variations in the system time.

### 3.1. Power Plant Specifications

The power plant that is the focus of this work is on the city of João Pessoa (Paraíba, Brazil), with an installed capacity of 340 MW and declared power for dispatch of 320 MW, the latter considered as maximum power. The operation of this plant is of the internal combustion type—that is, using thermoelectric to stationary combustion engines (which are commonly used in nautical applications for large ships).

The power plant contains 40 combustion engines, where 38 engines are type 18V32/40 of Korean manufacture and German design, and two engines are type 9L32/40 (of the same manufacture and design). Each engine is coupled to a 10 MVA generator, forming the motor-generator set, which has a delivery capacity of 8.7 MW at the generator’s output, a value that derives from the power delivered to the 9 MW motor shaft, minus the losses due to the coupling between the engine and generator.

To keep the engine running for long hours of operation, the plant uses a water cooling system that controls the temperature of the charge air (i.e., intake air after compression in the turbocharger), the temperature of the lubricating oil, and the temperature of the heads. Therefore, all process parameters must begin properly controlled, especially temperature parameters, as they directly contribute to the efficiency of the generating unit. To maintain these levels of control, according to the operating specifications, this cooling system consumes about 6 MW of energy to maintain its maximum power.

### 3.2. Water Cooling System

**Figure 1:**
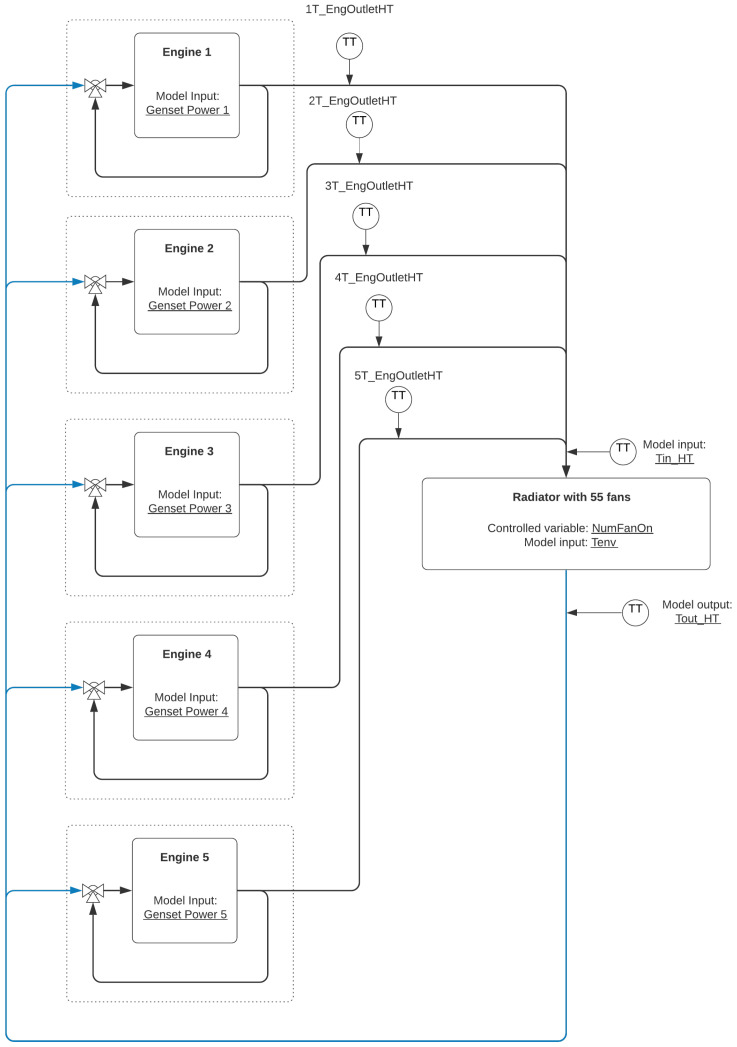
Water cooling system.

Normally, in thermoelectric plants with internal combustion engines, each engine has an individual cooling module, often composed of fans driven by frequency converters that facilitate the control of the fans by varying the rotation speed of the engines. However, the thermoelectric plant studied in this work presents a very particular system: it uses 55 fans to compose a radiator that is used to control the cooling water temperature of the five engines simultaneously, as shown in Figure 1. In this way, the radiator system seeks to stabilize the temperature to a reference of 62 °C. In addition, the fans are activated using a direct start configuration; that is, there is no possibility of fan speed variation. An important aspect of the water cooling system is that the determination of the number of radiators (*NumFanOn*) depends on the inlet temperature ($T_{in}\_HT$), the environmental temperature ($T_{env}$), and the observed outlet temperature value ($T_{out}\_HT$).

The simultaneous sharing of the refrigeration system with the five motors makes the characteristics of the process strongly nonlinear considering that the motors operate at different powers and present different problems, which have an impact on the operating temperature, in addition to the possibility of leaks in the circuit and, consequently, the entry of air impairing the heat exchange. On the other hand, the use of industrial controllers, such as proportional (P) and proportional-integral (PI) controllers, are inefficient in controlling this process throughout the operation, due to its strong nonlinear characteristic, requiring tuning of the parameters of the controller with some regularity, which is usually not feasible for the maintenance team.

**Figure 2:**
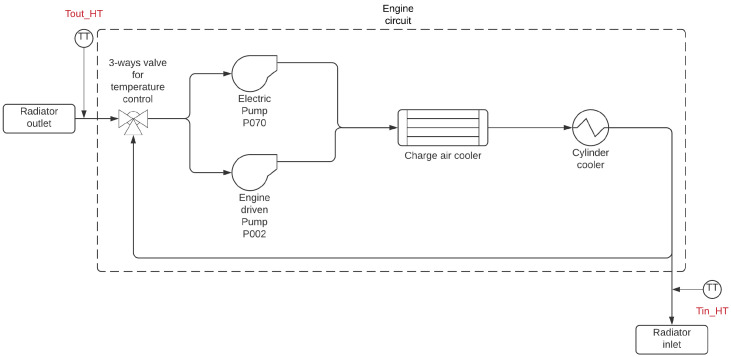
Temperature control system for cooling of an engine.

To control the water temperature, a proportional controller is currently used for each engine, which results in a controlled temperature $T_{in}\_HT$, as shown in Figure 2. In this figure, the manipulated variable is the angle of the three-way valve, whose operation takes the controlled variable to the set-point temperature. The position of the valve angle defines the energy transfer (in the form of heat) between the water supplied by the radiator and the water coming from the engine outlet, with temperature $T_{out}\_HT$.

This control strategy has some limitations, including: (a) zero error is not guaranteed in steady-state; (b) the nonlinearities of the system lead to a loss of tuning of the controller constant; and (c) the temperature control has a low performance due to external effects, mainly in rainy and night periods, where the decrease in the ambient air temperature is not considered to reduce the number of fans connected (i.e., used by the radiator), leading the cooling water system to consume a high amount of energy.

**Figure 3:**
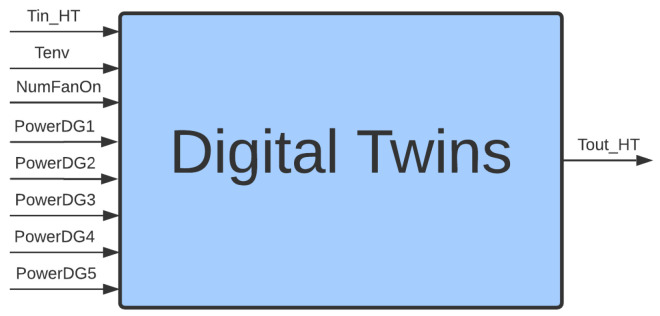
Digital Twins of cooling system.

The description of the water cooling system points to the need to develop Digital Twins, based on data and by analyzing the main variables that contribute to the radiator’s behavior, such that the input variables are synergistically incorporated into a single model: inlet water temperature in the high-temperature side radiator ($T_{in}\_HT$), environmental temperature ($T_{env}$), number of fans connected (NumFanOn), power of the Diesel Genset 1 (PowerDG1), power of the Diesel Genset 2 (PowerDG2), power of the Diesel Genset 3 (PowerDG3), power of the Diesel Genset 4 (PowerDG4), and power of the Diesel Genset 5 (PowerDG5). Furthermore, it should also consider the following output variable: the output water temperature in the high-temperature side radiator ($T_{out}\_HT$). This set of input and output variables was selected based on the knowledge of specialists—professionals who are in daily contact with the operations of the plant and equipment, and the existing control loop at the plant. Figure 3 illustrates the proposed Digital Twins, and the next section presents the methodology used to develop this model.

## 4. Digital Twins of the Cooling System

In this section, the steps of the proposed methodology to obtain the digital twins of the refrigeration system from the data stored in the plant’s supervisory system are presented.

The methodological approach for the development of the digital twins of the water cooling system is based on fuzzy set theory and the automatic rules extraction algorithm. Fuzzy logic was chosen, due to the possibility of extracting rules from a database of measurements of the refrigeration system, which are associated with the behavior of the real physical system. Another highlight of this methodology is the ability to generate new rules and update the knowledge bank when the system works in unknown situations; for example, when the model error exceeds an unwanted threshold. In this way, the developed digital twins will have an approximate dynamic response to the real physical system, and will follow the changes of the refrigeration system in real-time during its operation.

**Figure 4:**
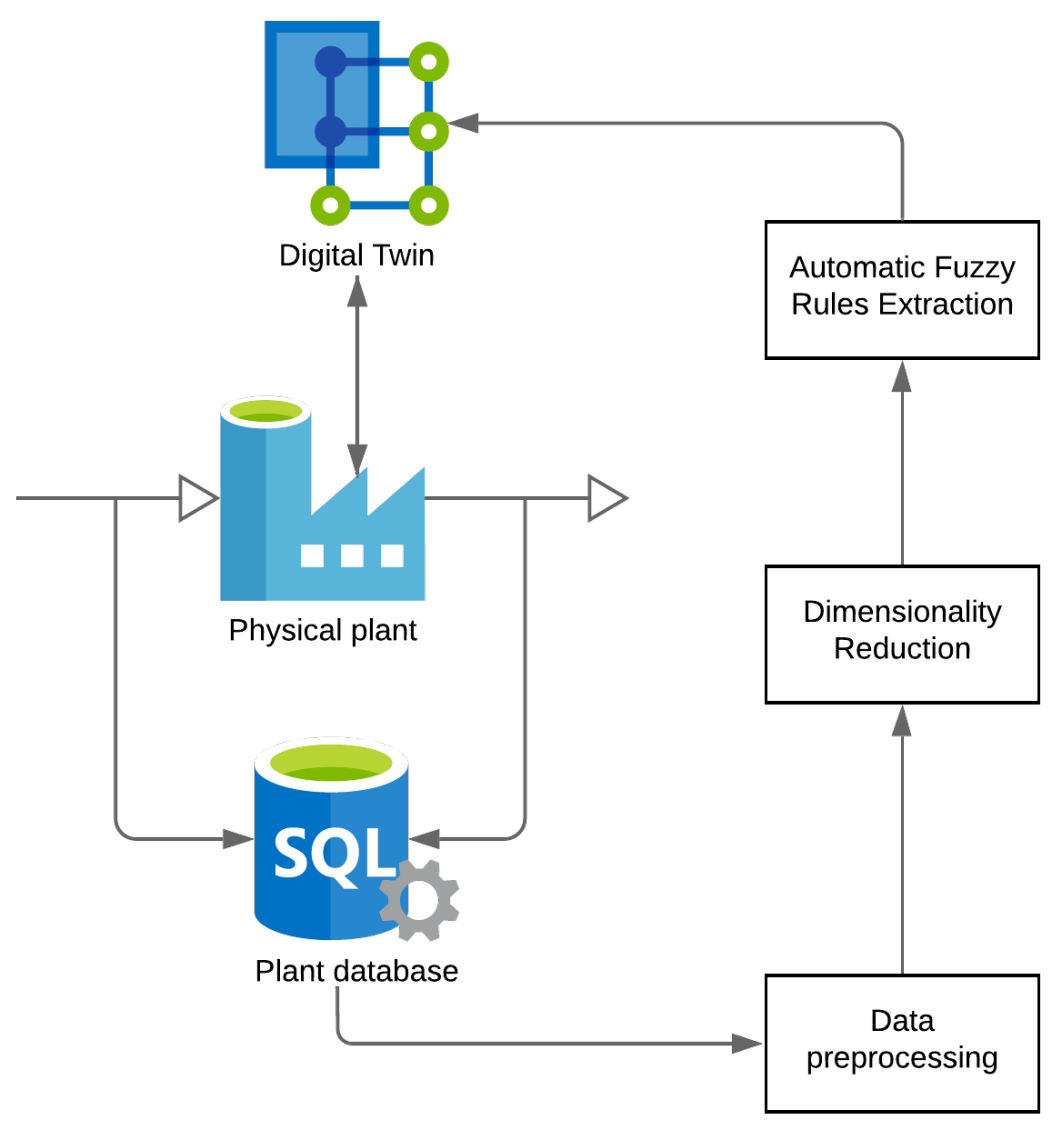
Block diagram of digital twins.

This approach has an advantage over solutions that are based on ANNs, where it is often necessary to have a training database with sufficiently many examples to build an approximate model of a system. Figure 4 illustrates the methodological procedure for the development of digital twins for the water cooling system. For the sake of clarity, the diagram is divided into four subsections.

### 4.1. Data Acquisition Systems

Due to the high intermittency in the dispatch of a power plant, the data stored in the bank contain a large amount of information that characterizes the nonoperating plant, which was not considered useful for the construction of the model, and must be removed before the construction of the digital twins of the cooling system. Thus, the database was constructed regarding the longest uninterrupted dispatch time of the plant in 2019, such that it contained the minimum amount of information relating to nonoperational plant data.

In this first stage, a data set was acquired with 1,120.200 samples of the variables $T_{in}\_HT$, $T_{env}$, *NumFanOn*, P1, P2, P3, P4, P5, and $T_{out}\_HT$, equivalent to the dispatch that took place between 14–25 May 2019, with a sampling time of one second for all variables.

### 4.2. Data Preprocessing

To ensure that rules were not generated from characteristics incompatible with the normal behavior of the system, it was necessary to preprocess the raw data to eliminate outliers, which can be generated by such events as connection failures between the sensors and the controller, breakage of sensor cables, or short-circuits in the sensor power supply.

**Figure 5:**
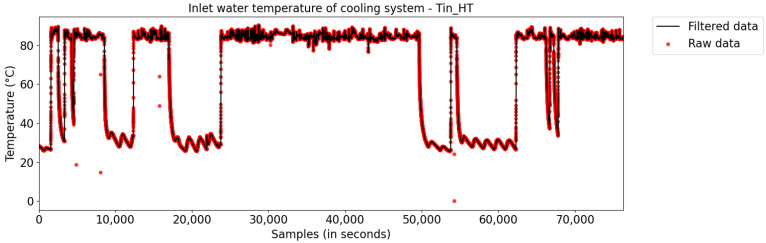
Pre-processing of the input temperature variable (Outliers).

In this step, the moving-average algorithm was used to eliminate outliers, by replacing them with neighboring samples. Figure 5 illustrates the result obtained after filtering the input temperature variable ($T_{in}\_HT$). In general, the mass of data contained in the plant’s database had few outliers, which was expected, as the data of all sensors are stored in the database using the PIMS (Plant Information Management Systems) software, which contains algorithms to handle the preprocessing and storage of data to record them more efficiently (both in terms of file size and quality).

**Figure 6:**
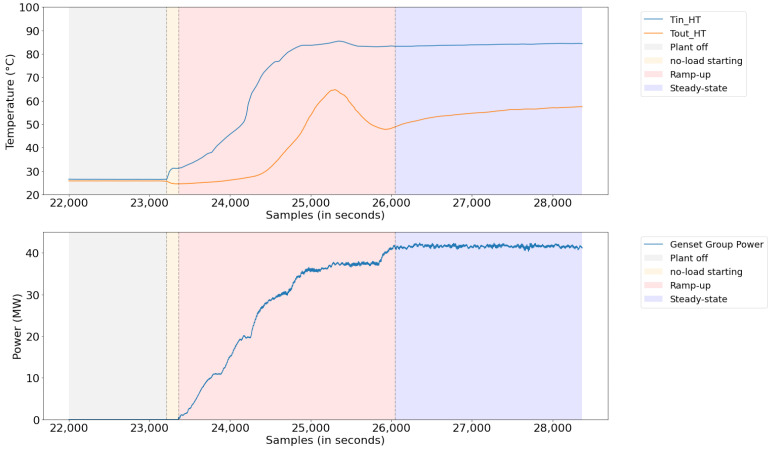
Inlet and outlet temperature in the radiator during pre- and poststart periods of plant.

Even when extracting the period with the longest continuous dispatch time of the plant, the data acquired contained characteristics of four different states of the plant: plant off, plant on start, plant on ramp (ramp up or ramp down), and steady-state. In Figure 6, these states are illustrated, from the data, at a time when the dispatch started. The data related to the stopped plant do not add value to the model, as there is no need to control the system temperature in this state; therefore, they were discarded. The data relating to the plant starting and ramping up or down contain characteristics related to the plant’s transient, where temperature control is more complicated due to the large temperature variation in the system, and the nonlinear characteristic of the system becomes more pronounced. The data related to the plant in steady-state is the state of the plant where the input and output variables are more stable, and the system has an attenuated nonlinear characteristic.

The last step of data preprocessing is to apply normalization to the inputs (i.e., transforming the data such that they have a mean value of zero and a standard deviation of 1). This is used for the next step, which involves reducing the number of variables based on principal component analysis (PCA) [28].

### 4.3. Reduction of Variables

Given the large number of digital twin variables (Figure 3), a methodology for reducing the dimensionality and optimizing the computational cost of the tool was considered necessary. For this purpose, we used the Principal Component Analysis (PCA) algorithm, which aims to reduce the dimensionality of a system to reduce the number of system variables without significant loss of information. This is possible because, in systems with many variables, redundant information is often expressed by different variables in the same process. By applying principal component analysis, it is possible to identify and reduce those variables with little expression in the system output.

**Figure 7:**
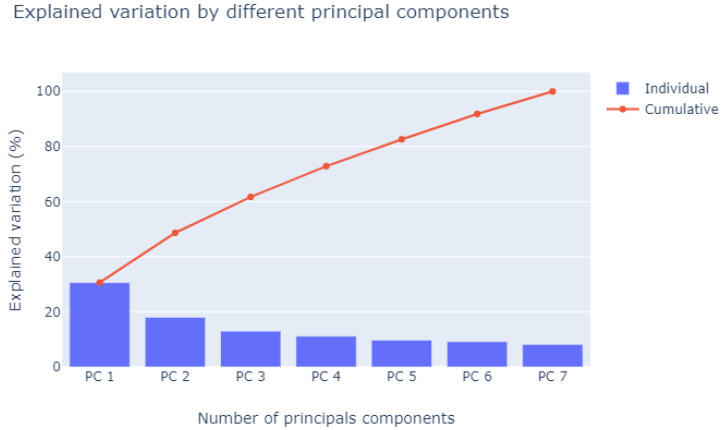
Variation explained by number of components used.

For application of PCA, the input representing the number of fans connected (NumFanOn) was discarded, as it is the manipulated variable of the refrigeration system; that is, it is used by the plant operator so the system can be stabilized at the desired temperature, thus carrying out an open loop control to search for the appropriate number of fans for that moment. Therefore, PCA was applied to the remaining seven inputs, reducing them to four inputs. This choice was made using the scree plot method (see Figure 7), which consists of analyzing the slope of the eigenvalues for each factor; in particular, locating the end of the slope and the beginning of the stabilization of the eigenvalues. In this figure, starting from PC4, the stabilization of the eigenvalues is noticeable [29]. Another method was used to confirm the choice of the four PCs: Jolliffe’s modification of Kaiser’s rule, which states that the PC cutoff can be chosen when the PC eigenvalue is equal to 0.7 [30]. In this case, the eigenvalues were [2.14 1.26 0.91 0.78 0.68 0.64 0.57], such that the PC4 eigenvalue was the last eigenvalue greater or equal to 0.7.

It is important to emphasize that variable reduction using PCA drastically decreases the interpretability obtained when using a fuzzy inference system as, when the reduction occurs, the physical variables captured by the sensors are transformed into principal components, which have no physical meaning. This causes the human understanding of the status of the samples to be lost. In the case of this work, this understanding becomes irrelevant, considering that the modeling sought is based on data and the main concern of the approach used is to achieve the smallest possible error with the least possible computational effort.

**Figure 8:**
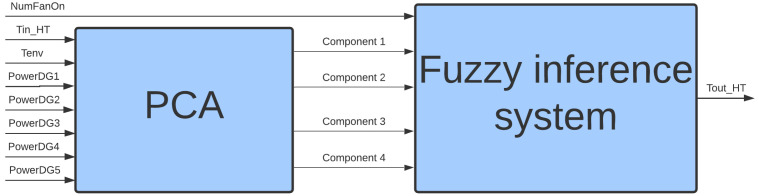
Digital twins input-output after dimensionality reduction.

After selecting and reducing variables, the digital twins of the water cooling system had five inputs and one output, as shown in Figure 8. This procedure had a direct impact on the number of rules generated from the database of measurements, being significantly lower for the model with five inputs.

**Figure 9:**
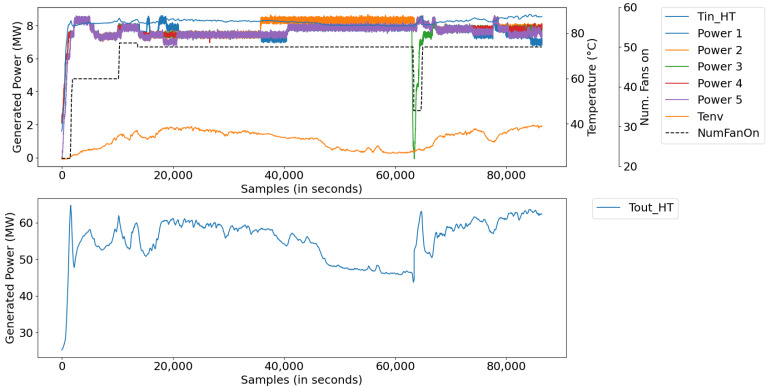
Cooling system measurements.

**Figure 10:**
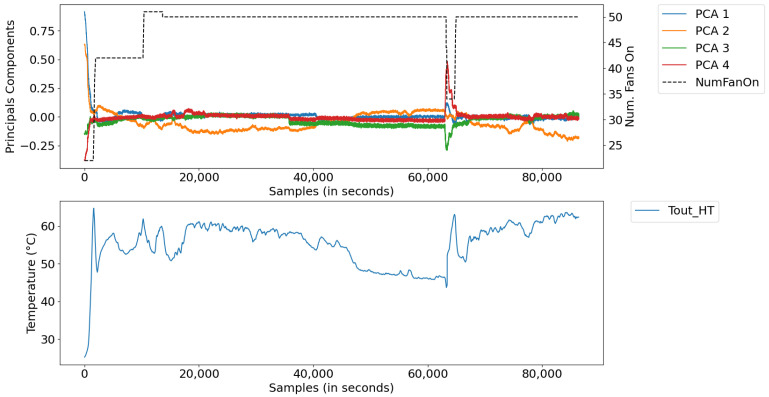
Number of fans, PCA components, and outlet temperature.

Figure 9 illustrates the measurements of the water cooling systems, corresponding to the model with eight inputs and one output (Figure 3). Figure 10 illustrates the coded inputs using the PCA, with five inputs and one output (Figure 8). In this last configuration, the measurements are used, by the automatic rule extraction algorithm, to build the knowledge base of the fuzzy inference system.

### 4.4. Automatic Extraction of Rules Using Fuzzy Logic

Following the methodology for the automatic extraction of fuzzy rules from numerical data presented in [14,15], we began with the definition of the linguistic variables and the division of the domains of each one using fuzzy sets of the triangular type. The linguistic variable NumFanOn has a domain of 0 to 55 fans connected, and 40 triangular fuzzy functions were used. The linguistic variables (PCA1, PCA2, PCA3, and PCA4) had domains in the range [−1,1], and 40 triangular fuzzy sets were used for each one. Finally, the linguistic variable $T_{out}\_HT$, which represents the outlet temperature, has a domain of 20–100 °C, and 40 triangular fuzzy functions were used.

A genetic algorithm was used to find the best number, position, and shape of triangular fuzzy sets. This approach was used with the aim to find the best configuration, which can decrease the computational effort and the minimum possible error.

Then, rules were generated for each set of input-outputs, as follows: “If (NumFanOn is V) and (PC1 is A) and (PC1 is B) and (PC1 is C) and (PC1 is D) then ( $T_{out}\_HT$ is T)”. For each set of input-output data, a fuzzy rule was generated, for a total of 86,400 rules. However, due to the dynamics of the refrigeration system, redundant rules were generated, which had to be identified and removed. Thus, of the 86,400 rules extracted initially, after treatment by the fuzzy rules bank, 1376 different rules were obtained.

As an example, this application was randomly extracted for a rule within the fuzzy rule knowledge database:

“IF (PCA1[comp20] AND PCA2[comp18] AND PCA3[comp18] AND PCA4[comp21] AND NumFanOn[fannum29]) THEN $T_{out}\_HT\left[temp17\right]$”.

This means that the inputs and outputs used in the automatic extraction algorithm in the creation of this rule activated the fuzzy set called “comp20” for PCA1, “comp18” for PCA2, “comp18” for PCA3, “comp21” for PCA4, “fannum29” for PCA2 and, in the output, the fuzzy set activated “temp17”.

After performing this procedure, the knowledge base of the fuzzy inference system was formed, which allowed for the construction of the digital twins of the refrigeration system. As the computational model follows the real-time behavior of the physical system, updates must be made to the rule bank for events that were not included in the original database. The following section describes the procedure used to update the fuzzy rules, which provides the digital twin with the ability to keep the cooling system model up to date.

### 4.5. Dynamically Updating the Knowledge Base

The concept of a digital twin consists of building a virtual model that can simulate physical assets for failure prediction, and to identify potential problems that the real physical system may have. For these purposes, the model must be capable of continually adapting to its physical twin [6].

Based on this characteristic of the digital twin, we proposed to develop a method to update the knowledge base dynamically to tune the model according to the result that was out-of-step with the real value of the process.

Normally, learning models from data is carried out by using a specific amount of data and looking for levels below an established metric, such as the mean square error. However, over time, the characteristics of the process plant are modified, due to the wear and tear of equipment, or the maintenance carried out in which parts are exchanged. Thus, the rules learned may become obsolete, especially if the training set comprises old data that do not reflect the current system dynamics.

Therefore, when the rule used to simulate the system output had an error greater than the result verified in the test bench, the rule was considered obsolete. Thus, an algorithm for updating rules in real-time was created to learn the new dynamics of the system featuring a digital twin.

The maximum instantaneous permissible error value of 5% was adopted, which represents the necessary trigger for the algorithm to create new rules to reduce the error. This adopted error value represents a variation of 3 °C of the radiator outlet temperature; a value considered acceptable by the plant specialist as, in the current control it uses a +3 °C dead band (i.e., it would not cause the triggering of alarms or unnecessary shutdowns if digital twins were used to help in controlling the number of fans). This value was adopted solely considering the knowledge of the plant specialist as, due to the environmental conditions in the region, the plant’s staff with experience in operating the plant on a day-to-day basis observed that such variation had no impact on the process.

**Figure 11:**
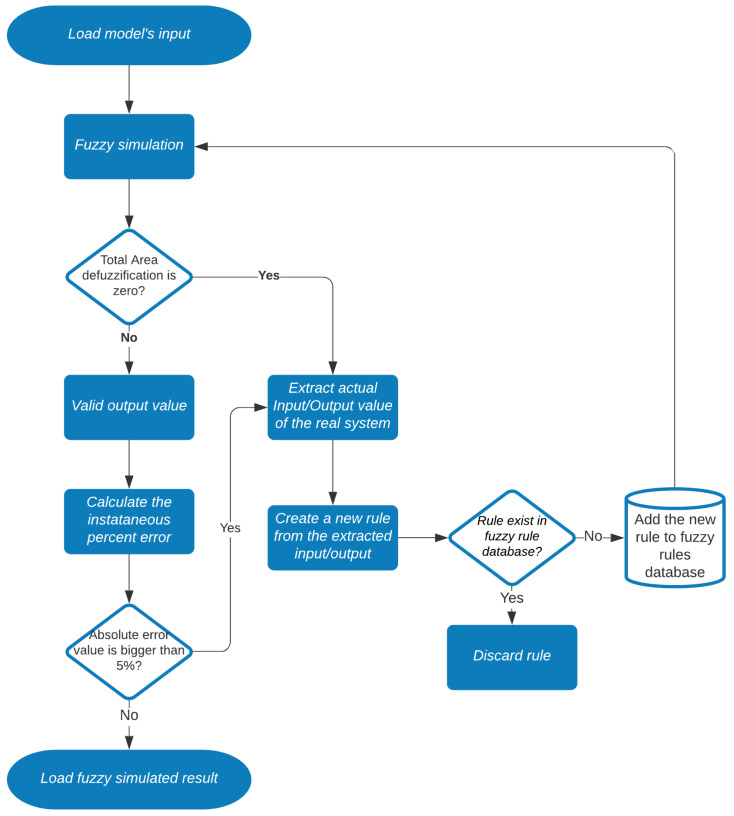
Flowchart of the fuzzy rules update algorithm.

Figure 11 illustrates the flowchart of the implemented algorithm. The algorithm verifies two important conditions for the model: (1) the output of the defuzzification block, when zero indicates invalid values (without activating rules); and/or (2) guaranteeing an error variation less than 5%. When these conditions present unsatisfactory responses, it is necessary to extract new rules from the scenario that generated the invalid condition and, thus, update the model without having to relearn the entire database. Another important point is that the algorithm allows you to insert rules (if they are unique) at any time, such as those obtained with respect to the knowledge of the industrial specialist.

### 4.6. Cooling System Fan Optimization Module

**Figure 12:**
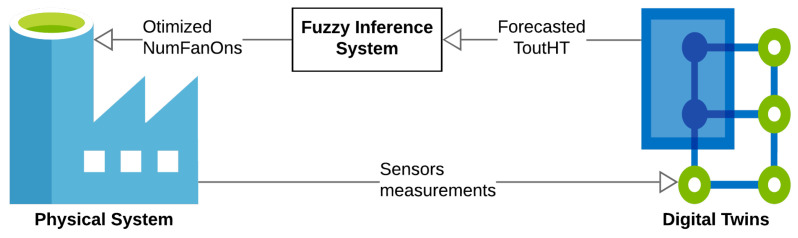
Application of digital twins to determine number of fans.

To increase the operational performance of the refrigeration system, an attempt was made to determine the number of fans in an optimized manner. For this, a fuzzy inference system was developed in conjunction with digital twins, as illustrated in Figure 12. This optimization made it possible to reduce the energy cost of the system and guarantee the proper functioning of the motors.

**Figure 13:**
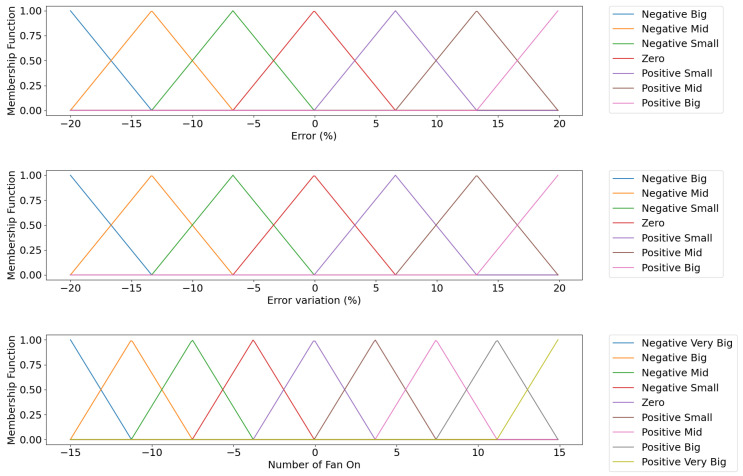
Fuzzy fan optimizer controller sets.

The fuzzy inference system is a temperature controller, whose set-point is the temperature defined according to the parameters stated by the manufacturer, being 62 °C. For this controller, the inputs are the error and the error variation, and the output is the variation in the number of fans, which must be used to update the number of fans. Figure 13 illustrates the fuzzy sets of variables in the inference system.

**Figure 14:**
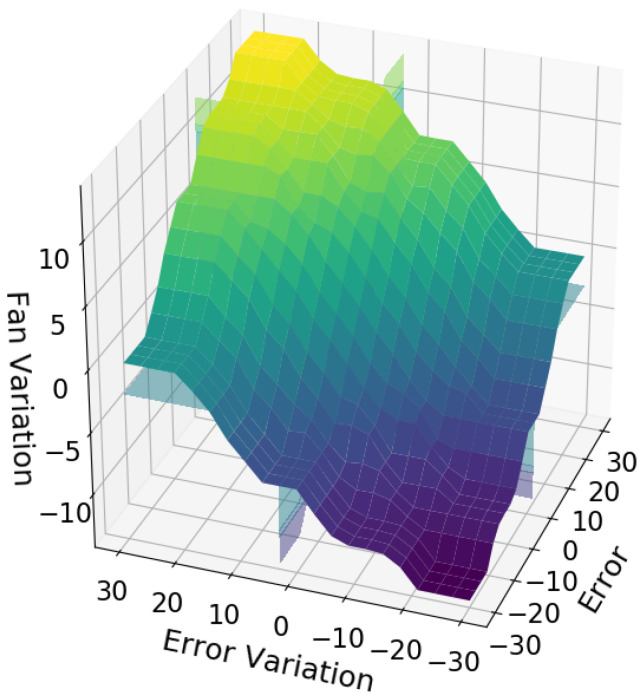
Surface of fuzzy inference system.

For the fuzzy inference system, 49 “If … Then” rules were created to cover all possible situations. Table 1 contains the 49 rules of the fuzzy optimizer (where PVB, positive very big; PB, positive big; PM, positive medium; PS, positive small; z, zero; NS, negative small; NM, negative medium; NB, negative big, and NVB, negative very big). Figure 14 illustrates the surface generated by the inference system, which reflects the ability to generate continuous values for variations in the number of fans.

## 5. Experimental Results

To validate the performance of the digital twins of the power plant’s water cooling system, in this section, our experimental results are presented. We considered three different scenarios: (a) performance of the digital twin operating during the permanent process regime; (b) performance of the digital twin during transients in the process (plant start and stop), and (c) digital twin performance during large transients, due to system control failure. Finally, the digital twins were used to determine the appropriate number of fans to be connected, by implementing a fuzzy inference system.

The training database was obtained from equipment installed in an internal combustion thermoelectric plant with the following specifications: hot water cooling system with 55 fans operated remotely, automatically, or manually (by plant operators), and five motor-generating units composed of MAN/STX 18V32/40 engines + 10MVA Hyundai generators. Such equipment was monitored using pt100 and thermocouple temperature sensors and transmitters.

The optimization of fuzzy sets is a complex task, because they are possible to optimize in several ways. We considered the number of fuzzy sets per variable, while the second parameter was the form of the fuzzy sets. In this work, all fuzzy variables had the same amount of fuzzy sets, which had triangular form.

In all scenarios presented, we considered a test database with 40,000 samples, equivalent to 11 h of uninterrupted operation. According to the plant specialist, it takes 1 h and 30 min for the process to achieve steady-state status; therefore, the period considered in each test was considered long enough to check the steady-state status of the digital twin.

### 5.1. Operation of the Model during Steady-State

**Figure 15:**
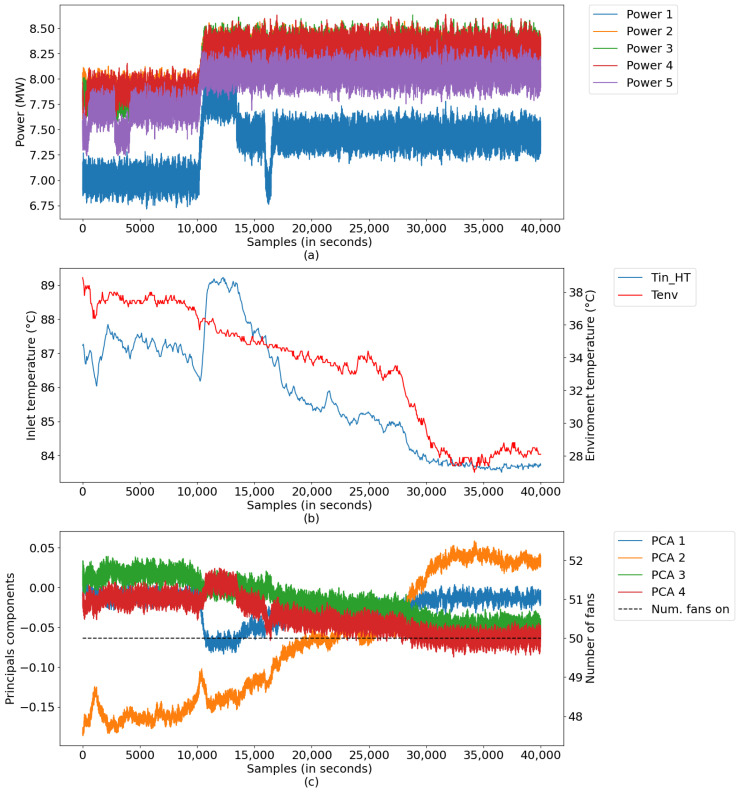
Process inputs: (**a**) powers of DGs; (**b**) $T_{in}\_HT$ and *environmental temp*, and (**c**) reduced inputs and NumFanOn.

In this scenario, digital twins were developed using data from a test database with a sampling period of 1 s, comprising a set of 40,000 samples, equivalent to a little more than 11 h of uninterrupted plant operation. The data refer to a generation in which there was no starting or stopping of the generating unit, only small changes in load. Figure 15 illustrates the measurements for this period, divided into: (a) power data of Diesel Gensets (DGs); (b) radiator inlet temperature and ambient temperature data, and (c) data converted into main components, along with the number of radiators.

**Figure 16:**
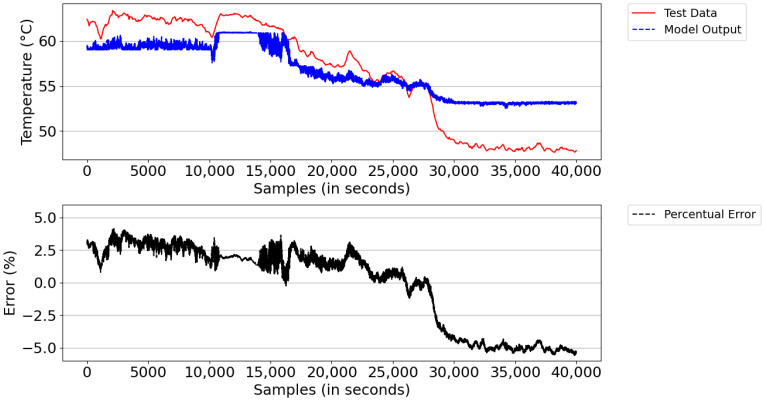
Model result versus actual $T_{out}\_HT$ value and instantaneous percentage error.

Figure 16 illustrates the result of the model simulation versus the actual system temperature value and the instantaneous percentage error—a parameter used to trigger the learning of new rules during the simulation. The model simulation started with the number of rules learned in the training database (i.e., 547 rules). After the simulation, the model detected learning opportunities and ended the period with 591 rules. The reason for learning was the lack of rules and points that reached above 5% instantaneous percentage error. From the experimental result, the digital twins showed an average percentage error of 5.1% and an absolute average error of 2.76 °C.

From the figure, both the outlet temperature of the cooling system dropped from 60 °C to below 55 °C in the sampling interval 25,000–35,000, where the power of the DGs remained constant and the number of fans turned on was equal to 50. This behavior was expected, given that the real system has a deficiency in temperature control; that is, it is unable to regulate the system’s outlet temperature when the ambient temperature decreases due to the change from day to night and/or when there are periods of rain.

### 5.2. Operation of the Model during the Plant Stop and Start Ramps

**Figure 17:**
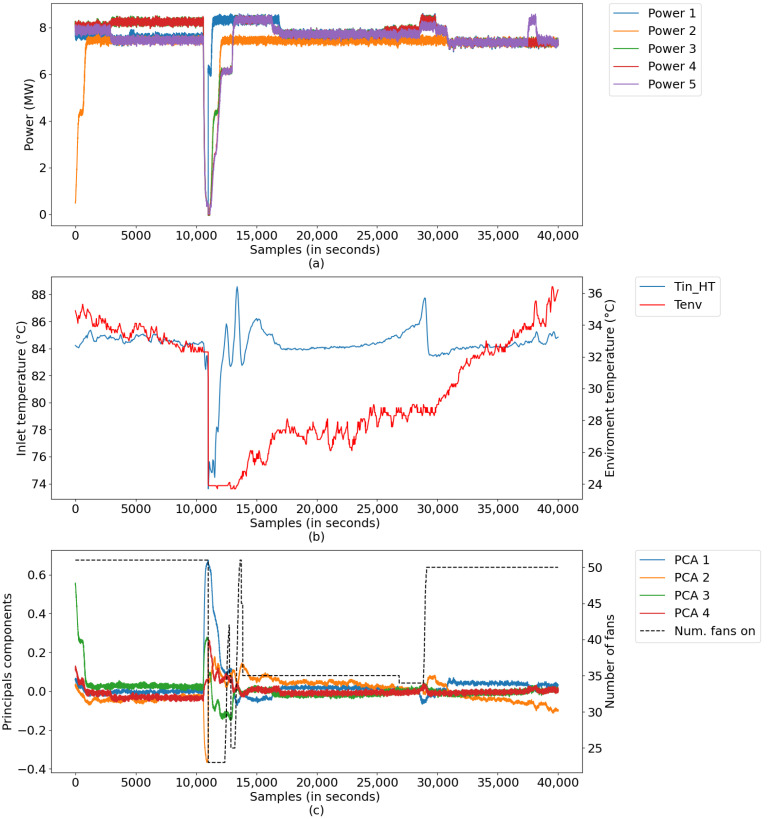
Process inputs: (**a**) powers of DGs; (**b**) $T_{in}\_HT$ and *environmental temp*, and (**c**) reduced inputs and NumFanOn.

In this scenario, the plant was scheduled to stop and start during the generation period. Figure 17 illustrates the measurements of this period, divided into (a) power data with DGs stop and start, (b) radiator inlet temperature and room temperature data, and (c) converted data main components, together with the number of radiators.

**Figure 18:**
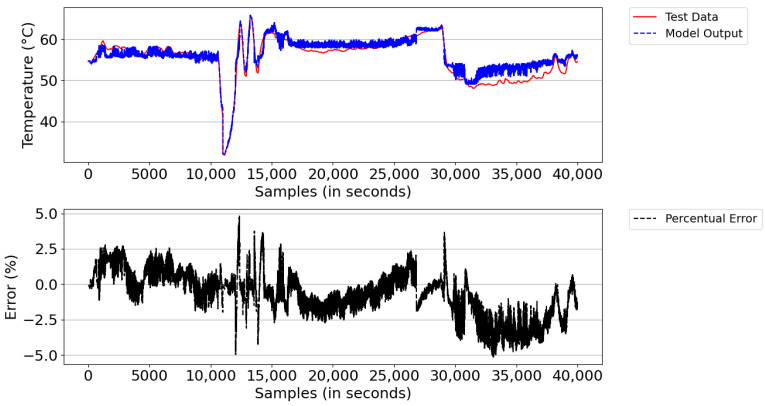
Model result versus actual Tout_HT value and instantaneous percentage error.

Figure 18 illustrates the result of approximation of digital twins, showing an average error of 4.41% and an absolute average error of 2.37 °C. In this figure, most of the time, the model presented an instantaneous percentage error below 5%. In the periods of transitions (stop ramp and climb ramp), it presented moments in which the error exceeded 5%, thus activating the trigger to learn new rules and reduce the error again. This happened because the digital twins initially did not learn the dynamics of ramping (moments of great transition). Due to the stop and start interval, the number of model rules increased from 591 to 754. Therefore, digital twins can learn the dynamics of the system, even in the face of new events, reinforcing their knowledge base about the real physical system.

The ability of digital twins to automatically extract new rules during operations became a feature related to great efficiency and importance, mainly for the self-adjustment of the model, keeping the error associated to the approach within the tolerable limits of operation.

**Figure 19:**
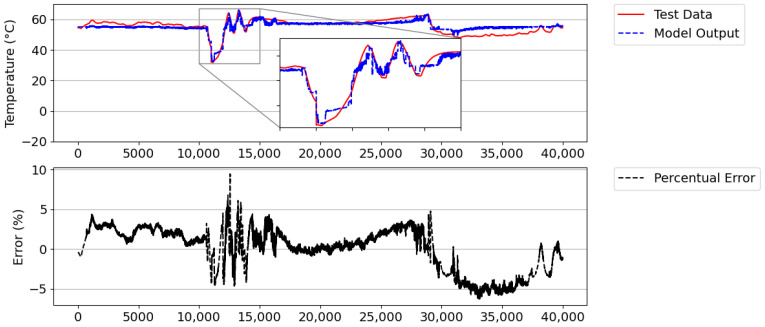
Result of model’s self-validation versus actual $T_{out\_HT}$ value and instantaneous percentage error.

Using digital twins with the new rules, a new simulation was performed using the same database (Figure 17) to validate the efficiency of the proposed dynamic learning method. In Figure 19, the results are illustrated, which show that an absolute mean error of 2.18 °C and an average percentage error of 4.07% were obtained. This figure indicates an improvement in performance in the stop and start ramp intervals of the DGs, with an error of less than 10%. This result was due to there already being relevant rules in the digital twin’s knowledge base, which allowed it to deal with this type of event.

### 5.3. Model Operation during Period with Transients

**Figure 20:**
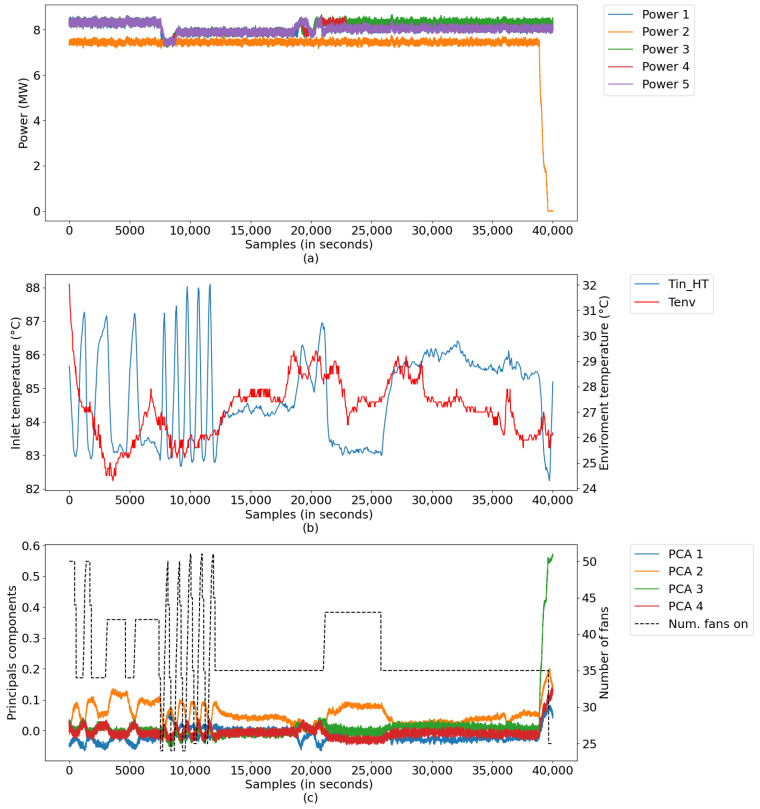
Process inputs: (**a**) powers of DG’s; (**b**) $T_{in}\_HT$ and *environmental temp*, and (**c**) reduced inputs and NumFanOn.

In this scenario, data were extracted from a period of operation of the plant, in which the system presented abnormal oscillations due to the constant shutdown/activation of a large group of fans—a product of the instability of the control system. Figure 20 illustrates the measurements, whose abnormal operating conditions of the system were found between samples 8000 and 12,000, characterized by: (a) power data, (b) radiator inlet temperature data and room temperature, and (c) data converted into major components, along with the number of radiators.

**Figure 21:**
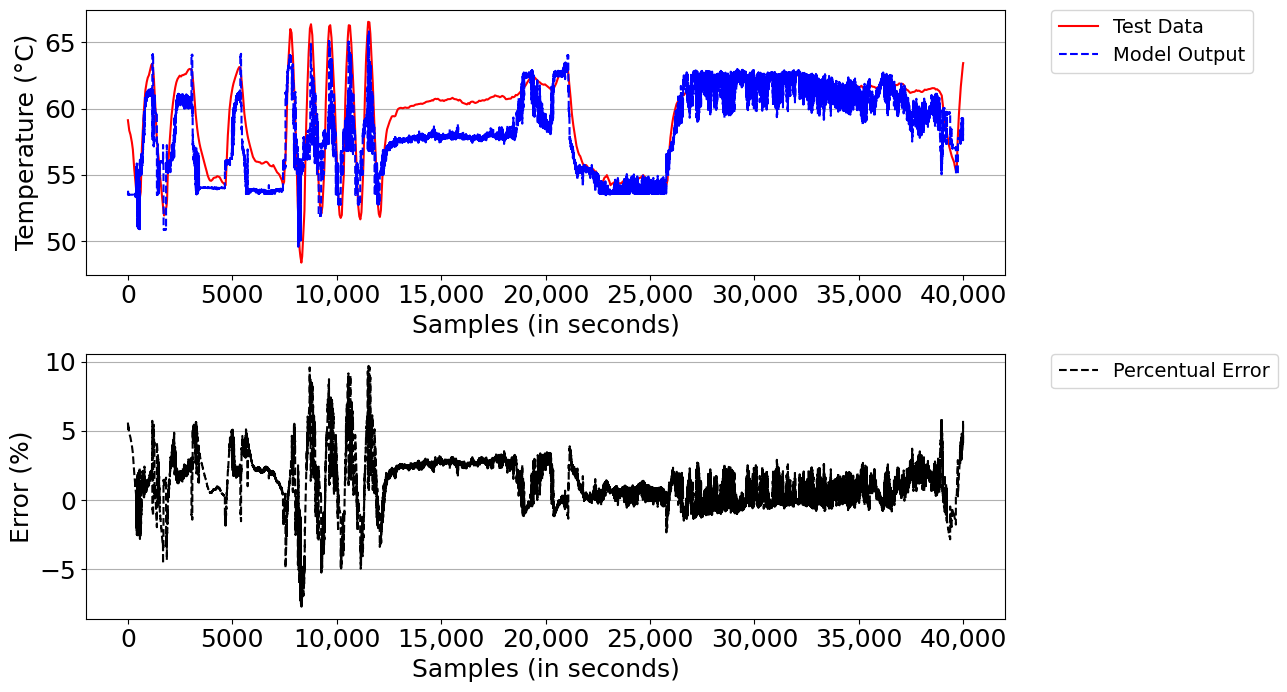
Model result versus actual $T_{out}\_HT$ value and instantaneous percentage error.

The digital twins performed well under conditions of instability, as illustrated in Figure 21. The instantaneous percentage error remained within the range of ±5% most of the time, only exceeding this value in a few situations. The model presented an average percentage error of 2.77% and 1.65 °C, showing high precision in the simulation of the system, even in abnormal situations.

This was because the model already underwent an experience like this situation; in this case, the stop/start ramp of the plant. That is, there were already rules (learned in scenario 2) that helped in the decision-making step in this period. Even so, after completing the simulation of the entire scenario, the model acquired a considerable amount of new rules, with a new total of 974 rules in the digital twin’s knowledge base.

### 5.4. Optimization of the Number of Fans Using Digital Twins

The optimization of the number of fans was performed considering the operation of the system during steady-state, the measurements of which are illustrated in Figure 15. The value used as a temperature reference at the radiator’s HT outlet was 62 °C, which was taken from the PID controller set-point in the generator units for the three-way valve at the engine’s water inlet.

**Figure 22:**
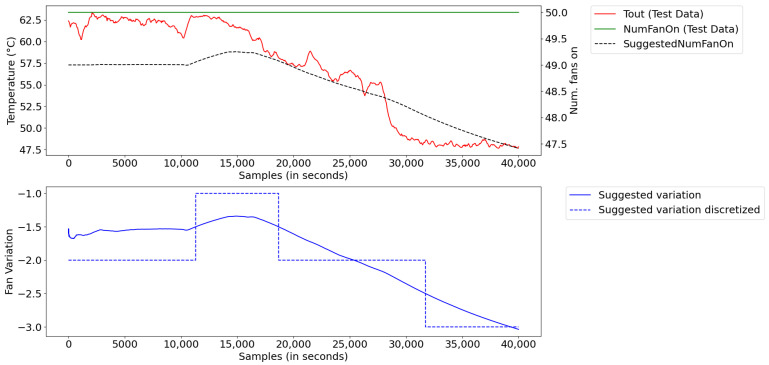
Optimization of number of fans for continuous operation.

Figure 22 illustrates the result for the optimization of the number of fans. For this, the inference system provides a suggestion of how many fans to turn off/on for the set-point to be reached. The fuzzy inference system provides continuous values; however, as it is not possible to turn the fans off or on in a fractional way, the result is rounded (and, thus, shown discretely). From this result, 50 fans did not need to be operating throughout the entire period, with a decrease of up to three fans.

A higher number of fans wastes energy in the thermal exchange. From the presented results, we verified that the system can operate most of the time with three less fans, accounting for about 1.44 MWh of saved energy per day per radiator.

## 6. Conclusions

In this work, a digital twin was developed for the water cooling system of a power plant to optimize the number of fans in the system.

The digital twin was developed based on a fuzzy inference system, whose knowledge base was generated using an automatic rule extraction algorithm and numerical data. In this model, an innovative functionality was implemented, which allows for new rules to be generated during the operation of the plant—that is, in the face of the occurrence of new events (e.g., starting ramp, stop ramp, transients due to shut down, and connection of radiators)—to control the temperature. Thus, during the plant’s operations, the rule bank is updated by the digital twins based on measurements from different scenarios, thus improving the model’s performance with each iteration.

In addition to learning the dynamic behavior of the real physical system, a digital twin was used to optimize the number of fans in the cooling system, through the use of a control system based on a fuzzy inference system.

From the experimental results, the error of approximation of the model had a satisfactory performance, presenting average percentage errors below 5% (i.e., below 3 °C).

This approach demonstrates that it is possible to create digital twins of a complete plant, and to create a plant simulator tool for training new control room operators and for experts to safely test the effect of changing parameters on the process. Furthermore, the greatest benefit presented by the presented technique is the ability to use a relatively small amount of data to create a model with low error response; thus, it could be possible to modernize older industrial plants more easily, given that such plants usually do not have a large amount of stored production process data.

## Figures and Tables

**Table 1:** System rules.

E\ΔE	NB	NM	NS	Z	PS	PM	PB
NB	NVB	NB	NB	NM	NM	NS	Z
NM	NB	NB	NM	NM	NB	Z	PS
NS	NB	NM	NM	NS	Z	PS	PM
Z	NM	NM	NS	Z	PS	PM	PM
PS	NM	NS	Z	PS	PM	PM	PB
PM	NS	Z	PS	PM	PM	PB	PB
PB	Z	PS	PM	PM	PB	PB	PVB

## Abbreviations

The following abbreviations are used in this manuscript:

DG	Diesel Genset
SCADA	Supervisory Control and Data acquisition
PID	Proportional–Integral–Derivative controller
GAs	Genetic Algorithms
LT	Low Temperature
HT	High Temperature
NumFanOn	Number of Fans connected
$T_{in}\_HT$	Reference Temperature at the radiator’s HT inlet
$T_{out}\_HT$	Reference Temperature at the radiator’s HT Outlet
ANN	Artificial Neural Network
SVM	Support Vector Machine
VIA	Validity Internal Analysis

## References

[1] Almeida C.F., Maciel V.G., Cybis L.F.A. (2017). Global warming potential assessment for operation of thermoelectric power plant in Manaus. Revista Latinoamericana en Análisis de Ciclo de Vida (LALCA).

[2] Saha B., Patel V., Chaterjee K. Assessment of process parameter to improving power plant performance. Proceedings of the Innovative Applications of Computational Intelligence on Power, Energy and Controls with Their Impact on Humanity (CIPECH/IEEE).

[3] Kruhol M., Lasurenko O., Vanin V., Tomashevskyi R. Group Regulation Efficiency Analysis for Thermal Power Plant Auxiliaries. Proceedings of the IEEE 6th Int. Conf. on Energy Smart Systems (ESS).

[4] Lei Z., Zhou H., Hu W., Liu G.P., Guan S., Feng X. (2021). Towards a Web-Based Digital Twin Thermal Power Plant. IEEE Trans. Ind. Inform..

[5] Ji H., Li J., Zhang S., Wu Q. Research on Water Resources Intelligent Management of Thermal Power Plant Based on Digital Twins. Proceedings of the IEEE 6th International Conference on Cloud Computing and Big Data Analytics (ICCCBDA).

[6] Farsi M., Daneshkhah A., Hosseinian-Far A., Jahankhani H. (2020). Digital Twin Technologies and Smart Cities.

[7] Elsisi M., Mahmoud K., Lehtonen M., Darwish M.M.F. (2021). Reliable Industry 4.0 Based on Machine Learning and IoT for Analyzing, Monitoring, and Securing Smart Meters. Sensors.

[8] Qi Q., Tao F. (2018). Digital Twin and Big Data Towards Smart Manufacturing and Industry 4.0: 360 Degree Comparison. IEEE Access.

[9] Tao F., Zhang H., Liu A., Nee A.Y.C. (2018). Digital Twin in Industry: State-of-the-Art. IEEE Trans. Ind. Inform..

[10] Chen X., Guan T. Research on the Predicting Model of Convenience Store Model Based on Digital Twins. Proceedings of the ICSGEA 2018-International Conference on Smart Grid and Electrical Automation.

[11] Xu Y., Sun Y., Liu X., Zheng Y. (2019). A Digital-Twin-Assisted Fault Diagnosis Using Deep Transfer Learning. IEEE Access.

[12] Sivalingam K., Sepulveda M., Spring M., Davies P. A Review and Methodology Development for Remaining Useful Life Prediction of Offshore Fixed and Floating Wind turbine Power Converter with Digital Twin Technology Perspective. Proceedings of the ICGEA 2018-International Conference on Green Energy and Applications.

[13] Antonelli M., Ducange P., Marcelloni F., Segatori A. (2016). On the influence of feature selection in fuzzy rule-based regression model generation. Inf. Sci..

[14] Wang L.-X., Mendel J.M. (1992). Generating Fuzzy Rules by Learning from Examples. IEEE Trans. Syst. Man Cybern..

[15] Dutu L.C., Mauris G., Bolon P. (2018). A Fast and Accurate Rule-Base Generation Method for Mamdani Fuzzy Systems. IEEE Trans. Fuzzy Syst..

[16] Cordon O., Herrera F., Villar P. (2001). Generating the knowledge base of a fuzzy rule-based system by the genetic learning of the data base. IEEE Trans. Fuzzy Syst..

[17] Bajpai P., Kumar M. (2010). Genetic algorithm—An approach to solve global optimization problems. Indian J. Comput. Sci. Eng..

[18] Tan S.C., Lim C.P. (2004). Application of an Adaptive Neural Network With Symbolic Rule Extraction to Fault Detection and Diagnosis in a Power Generation Plant. IEEE Trans. Energy Convers..

[19] Fu L.M. (1991). Rule learning by searching on adapted nets. Proceedings of the 9th National Conference on Artificial Intelligence.

[20] Fu L.M. (1994). Rule generation from neural networks. IEEE Trans. Syst. Man Cybern..

[21] Towell G., Shavlik J. (1993). The extraction of refined rules from knowledge based neural networks. Mach. Learn..

[22] Thrun S.B. (1993). Extracting Provably Correct Rules from Artificial Neural Networks.

[23] Tickle A.B., Orlowski M., Diederich J. (1994). DEDEC: Decision Detection by Rule Extraction from Neural Networks.

[24] Bondarenko A., Aleksejeva L. (2018). Methodology for Knowledge Extraction from Trained Artificial Neural Networks. Inf. Technol. Manag. Sci..

[25] Castro J.L., Flores-Hidalgo L.D., Mantas C.J., Puche J.M. (2007). Extraction of fuzzy rules from support vector machines. Fuzzy Sets Syst..

[26] Chaves A.C.F., Vellasco M.M.B.R., Tanscheit R. (2013). Fuzzy rules extraction from support vector machines for multi-class classification. Neural Comput. Appl..

[27] Lermer M., Reich C. Creation of Digital Twins by Combining Fuzzy Rules with Artificial Neural Networks. Proceedings of the IECON 2019-45th Annual Conference of the IEEE Industrial Electronics Society.

[28] Shlens J. (2014). A Tutorial on Principal Component Analysis. arXiv.

[29] Kaiser H.F. (1958). The varimax criterion for analytic rotation in factor analysis. Psychometrika.

[30] Jolliffe I.T. (2002). Principal Component Analysis.

